# Peer Microaggressions and Social Skills among School-Age Children of Sexual Minority Parents through Assisted Reproduction: Moderation via the Child–Teacher Relationship

**DOI:** 10.1007/s10964-022-01588-3

**Published:** 2022-03-11

**Authors:** Nicola Carone, Eleonora Innocenzi, Vittorio Lingiardi

**Affiliations:** 1 Department of Brain and Behavioral Sciences, University of Pavia, Piazza Botta 11, 27100 Pavia, Italy; 2 Department of History, Culture and Society, “Tor Vergata” University of Rome, Via Columbia 1, 00133 Rome, Italy; 3 Department of Dynamic and Clinical Psychology, and Health Studies, “Sapienza” University of Rome, Via degli Apuli 1, 00185 Rome, Italy

**Keywords:** Microaggressions, Child–teacher relationship, Social skills, Sexual minority parents, Assisted reproduction

## Abstract

The impact of peer microaggressions and the child–teacher relationship on the social skills of children with sexual minority parents has received little attention. The current study used a mixed-method, multi-informant, two-wave longitudinal design to address this research gap. Thirty-seven children of lesbian mothers through donor insemination and 33 children of gay fathers though surrogacy (wave 1: *M*_*age*_ = 8.3 years, *SD* = 1.6; 51.4% female; wave 2: *M*_*age*_ = 9.9 years, *SD* = 1.7), all school-aged and residing in Italy, participated together with their 140 parents and 55 teachers. Approximately two-thirds of the children reported at least one peer microaggression and, on average, microaggressions were of a low intensity. Child–teacher relationships were of high quality (i.e., characterized by high safe haven–seeking and secure base use, and low conflict). Both parents and teachers reported high levels of child social skills. However, more intense W1 microaggressions predicted lower W2 social skills among children reporting a lower W1 child–teacher relationship quality, and greater W2 social skills among those reporting a higher W1 child–teacher relationship quality. These results support the child–teacher relationship as a potentially secure context in which children can “mentalize” negative experiences such as microaggressions and improve their social skills. In this vein, considering microaggression, attachment, and developmental intergroup theories, teachers must attune to the school experiences of children with sexual minority parents and cultivate caring classroom environments that are sensitive to family diversity.

## Introduction

In the elementary school setting, children of sexual minority parents through assisted reproduction are at significant risk of experiencing peer microaggressions—a form of implicit stigmatization comprising brief and commonplace daily verbal, behavioral, or environmental indignities (Sue et al. 2007)—due to prejudicial beliefs and attitudes towards groups that are perceptually salient and/or proportionally distinct. Such microaggressions may arise in the form of peers’ insensitive questions or ambiguous comments with respect to the children’s family structure (i.e., their parents’ sexual orientation and/or their assisted conception background), similar to what has been documented for adopted children of sexual minority parents (Farr et al. 2016a, Farr et al. 2016b, Garber & Grotevant 2015). Given the subtle and daily nature of many microaggressions, from an attachment perspective, it is crucial that microaggressed children perceive warm relationships with teachers. More specifically, they must consider teachers reliable adults to whom they can turn when upset or worried and use relationships with teachers as secure bases from which to comfortably explore their learning and social environments.

Very little is known about both the peer microaggression experiences of children of sexual minority parents through assisted reproduction and the role of child–teacher relationship quality in reducing or amplifying the negative impact of these microaggressions on children’s social relationships with peers in the school setting. The current study drew on microaggression (Sue & Spanierman 2020), attachment (Bowlby 1988, Verschueren 2015), and developmental intergroup (Bigler & Liben 2006) theories to longitudinally investigate the moderating role of child–teacher relationship quality in the association between experiences of microaggression and social skills among school-age children of lesbian mothers through donor insemination and gay fathers through surrogacy in Italy. The results contribute to the growing body of evidence indicating that experiences of microaggression, especially when chronic and long-term, can be detrimental for child psychosocial adjustment (Bos et al. 2021, Carone et al. 2018, Carone et al. 2021a, Farr et al. 2016a, Farr et al. 2016b). They also call teachers to cultivate caring classroom environments that are sensitive to family diversity.

### The Study Context

Although recent decades have seen increased societal acceptance of non-traditional parents (e.g., parents representing diverse genders and sexual orientations), greater activism in support of the civil rights of sexual minority groups, and more inclusive access to assisted reproduction (Pew Research Center 2020), in Italy, sexual minority people still face significant challenges in their efforts to form a family. Access to adoption and domestic assisted reproduction is prohibited to them. Also, since coparenting arrangements do not generally represent a preferred path to parenthood, most Italian sexual minority couples who desire children must turn to cross-border reproductive services in countries where donor insemination and surrogacy are offered to non-residents, irrespective of their sexual orientation, gender identity, and marital status (Carone et al. 2017a, Lingiardi & Carone 2016a, Lingiardi & Carone 2016b, Lingiardi et al. 2016).

Research conducted on Italian sexual minority parent families has generated findings that align with evidence produced in other sociocultural contexts (e.g., the United States, the United Kingdom, the Netherlands; for reviews, see Bos & Gartrell 2020, Fedewa et al. 2015, Golombok 2020, Patterson 2017, Tasker 2005), indicating that the parents are as competent and well-adjusted as different-sex parents and that their children demonstrate healthy development (Baiocco et al. 2018, Carone et al. 2018, Carone et al. 2020a, Carone et al. 2020b, Carone et al. 2021a, Carone et al. 2021b). Studies have also indicated that sexual minority families continue to be challenged by stigmatization (Ioverno et al. 2018, Lingiardi et al. 2020, Pistella et al. 2018), which can reduce parents’ parenting quality and have negative consequences for children’s psychosocial functioning (Carone et al. 2017b, Carone et al. 2018). However, much remains unknown about the experiences of peer stigmatization among school-age children of sexual minority parents in the Italian setting, and the implications of such experiences on the children’s social skills.

### Microaggression Experiences of Children of Sexual Minority Parents through Assisted Reproduction

While early theorizing on microaggression focused on racial/ethnic targeting (i.e., towards African American individuals), microaggressions can in fact be expressed towards any marginalized group (e.g., women, religious minority groups, sexual and gender minority people) (Sue & Spanierman 2020), including sexual minority parent families. This is particularly true in more conservative contexts, such as Italy, where both explicit and implicit forms of marginalization, exclusion, and stigma against sexual minority parent families persist, often embedded in societal institutions (e.g., legal and school systems; Baiocco et al. 2020, Ioverno et al. 2018).

Microaggressions can manifest as microassaults, microinsults, or microinvalidations (Sue & Spanierman 2020). Microassaults represent the most overt form of microaggression and include conscious, deliberate, and either subtle or explicitly biased attitudes, beliefs, or behaviors that are communicated to marginalized groups through environmental cues, verbalizations, or behaviors. For children of sexual minority parents, an example of a microassault would be derogatory name-calling (e.g., “I was told I was going to hell because I had two moms”; Farr et al. 2016a, p. 91). Microinsults are characterized by verbal and non-verbal interpersonal exchanges that convey stereotypes, rudeness, and insensitivity and that demean a person’s marginalized identity. A microinsult towards a sexual minority parent family would be the stereotyping of all children of lesbian or gay parents as, themselves, non-heterosexual. Finally, microinvalidations include both verbal and non-verbal interpersonal communications that exclude, negate, or nullify the thoughts, feelings, or experiential reality of a marginalized group. An example would be the repeated questioning of children of gay fathers through surrogacy as to the whereabouts of their mother.

A recent review of the occurrence and impact of stigmatization—including microaggressions—on the daily lives of sexual minority parent families noted conflicting evidence on whether children of sexual minority parents experience higher or similar rates of teasing than children of heterosexual parents (Bower-Brown & McConnachie 2020). However, regardless of the amount, the content of the teasing appears to differ when it targets children of sexual minority parents relative to children of heterosexual parents, insofar as the former’s family structure (e.g., not having a father/mother, having two mothers/fathers, having a lesbian mother/gay father) can represent an additional and unique hook for the abuse.

Previous studies have indicated that, while preschool-age children rarely experience teasing due to their parents’ non-heterosexual orientation (Gartrell et al. 2000), such experiences become increasingly common as children reach school age and enter adolescence (Gartrell et al. 2005, Kosciw & Diaz 2008, van Gelderen et al. 2012). This may be explained by the development of prejudicial views in the elementary school years, as predicted by developmental intergroup theory (Bigler & Liben 2006), which assumes that children are active agents in the creation of prejudices and especially likely to develop prejudice towards groups that are perceptually salient and/or proportionally distinct. Also, in middle childhood (i.e., ages 6–12 years), children are better equipped to understand and articulate potentially microaggressive experiences enacted by peers, given their increasingly complex cognitive sophistication and refined language skills (Eccles 1999). Indeed, a previous study examining peer aggression, victimization, bullying, and teasing among school-age children found that the children were more accurately and reliably able to report their victimization experiences than were their parents, teachers, and peers (Ladd & Kochenderfer-Ladd 2002).

By middle childhood, children are also able to understand and articulate possible reasons for their victimization (Visconti et al. 2013a) and to differentiate between the harms caused by physical bullying versus emotional teasing (Harwood & Copfer 2015). Finally, between the ages of 6–10 years, children improve their ability to infer individuals’ stereotypes and become more aware of broadly held stereotypes (McKown & Weinstein 2003). Overall, these results complement the findings of previous studies with children from diverse families (e.g., single-father families, gay father surrogacy families, adoptive lesbian mother families) emphasizing that, by the age of 6–8 years, children begin to grasp the significance of their conception and the potential impact of their family arrangement on the wider social context, including their peers (e.g., Carone et al. 2021a, Farr et al. 2016a, Messina & Brodzinsky 2020). Based on these developmental considerations and previous evidence, it can therefore be expected that school-age children in this sample may be similarly able to accurately describe experiences of victimization and marginalization in an interview setting, including those that are characterized by subtle microaggressions.

Although having sexual minority parents is not, in itself, a risk factor for the development of psychological problems (e.g., Baiocco et al. 2018, Carone et al. 2018, Farr 2017, Gartrell et al. 2018, Golombok 2020), school-age children who are stigmatized due to their family structure are more likely to have lower self-confidence and more absences from school, and to exhibit more behavioral problems (Bos & Gartrell 2020, Bos & van Balen 2008, Carone et al. 2018, Kosciw & Diaz 2008). In the United States, a study with sexual minority parent families overtly framed by microaggression theory examined the behavioral adjustment and school experiences of 49 school-age adopted children (*M*_*age*_ = 8 years; 47% female) in 22 two-mother and 27 two-father families (Farr et al. 2016b). Although only 8% of the parents reported that their children were teased or bullied for having sexual minority parents, the children who perceived more microaggressions were reported by their parents and teachers as displaying more problem behaviors.

In the Netherlands, low levels of stigmatization were found among 63 8- to 12-year-old children of lesbian mothers (Bos & van Balen 2008). Despite this, boys more often reported that their peers excluded them because of their non-traditional family structure, while girls more often reported that their peers gossiped about the fact that they had two lesbian mothers. In another study involving a sample of 76 10-year-old children of lesbian mothers in the United States, 43% reported having been victims of homophobia and 69% reported negative feelings about these experiences (Bos et al. 2008). Similarly, a national United States sample of grade school children (i.e., kindergarten through grade 12) found that 40% of children with sexual and gender minority parents reported experiences of harassment and 23% reported feeling unsafe at school due to their family structure (Kosciw & Diaz 2008). Some also reported harassment due to their peers’ assumptions or perceptions about their non-heterosexual orientation. Similarly, 23% reported mistreatment and negative remarks from their peers’ parents, as a result of having sexual and gender minority parents. Finally, some evidence is available that experiences of stigmatization among adolescent children of lesbian parents through donor conception have adverse consequences for the development of problem behaviors in emerging adulthood (Bos et al. 2021).

Notwithstanding these results, research on microaggressions has been critiqued (for a detailed discussion, see Lilienfeld 2017, Sue 2017) for, among other reasons, relying exclusively on respondents’ subjective reports. This method of assessment may, in fact, result in both the underestimate of microaggressions (given the social undesirability of admitting one has perpetrated microaggressions) and the overestimate of microaggressions (given minority group members’ tendency to misinterpret ambiguous behaviors or statements as potentially microaggressive). A further criticism holds that the assessment of microaggressions through self-report might facilitate that respondents’ personality traits, such as negative emotionality, color their interpretation of items (Lilienfeld 2017). Considering these criticisms, the current study explored microaggressions through a semi-structured interview administered to children. Two independent, external coders reviewed the interview transcripts to identify microaggressions and evaluate their intensity, based on a codebook for assessing the impact of microaggressions on the lives and behavioral adjustment of adopted children of sexual minority parents (Farr et al. 2016a, Farr et al. 2016b).

### Links Between Child Social Skills and Child–Teacher Relationship Quality in Middle Childhood

In addition to the cognitive and linguistic skills mentioned above, further developmental and relational tasks are mastered in middle childhood, as children experience increasingly complex interactions, begin to compare their performance with that of their peers (who may validate or question their abilities), and are confronted with a variety of family forms. In this vein, social skillfulness is a multi-dimensional construct that defines a series of competencies that children acquire in middle childhood, in relation to their interactions with peers (Collins & Madsen 2016). These competencies are learned behaviors that combine cognitive and interpersonal skills, and they allow the child to develop healthy relationships while simultaneously sharing, helping, and regulating their character and temperament (Berry & O’Connor 2010).

During this period, entry into a peer group is fundamental for the development of social skills. For this, the ability to make contacts and receive acceptance from peers is key. Children also develop more effective reasoning skills, and their descriptions of themselves and others achieve greater stability and depth; they also acquire conflict resolution strategies aimed at maintaining social relationships with peers, and they can more readily recall information that can be used to manage new situations or to solve problems (Collins & Madsen 2016). Awareness of family diversity, acceptance, and conflict resolution strategies is especially relevant for children of sexual minority parents, whose daily experiences at school may be heteronormative (e.g., celebrating Mother’s Day or Father’s Day). In this context, they are likely to be microaggressed by peers in the form of questions (e.g., “Where is your dad?” “Why do not you have a mom?”) and heterosexist comments (e.g., “That’s so gay”) (Farr et al. 2016a, Haines et al. 2018, Nadal 2013).

Although the literature indicates that children of sexual minority parents generally have effective coping strategies to deal with microaggressions, develop positive perceptions of their family, and navigate experiences of difference with resilience (Farr et al. 2016a)—due in part to their ability to ascribe meaning to their own and others’ behaviors and their ability to take on the perspectives of others—the cumulative effect of these microaggressions is likely to be detrimental. Research has investigated several domains (e.g., depression, access to healthcare, sensitive parenting, suicidal ideation) in which microaggressions may exert a negative impact on sexual minority individuals and their families (Carone et al. 2021b, Kaufman et al. 2017, Sue & Spanierman 2020). However, evidence is lacking on whether—and to what extent—microaggressions influence social skills among school-age children of sexual minority parents.

In middle childhood, most microaggressions are experienced at school (Turner et al. 2011). Thus, teachers may play a key role in shaping the general peer ecology and supporting students’ social development, by imparting information, socializing appropriate interpersonal behavior, and providing guidance and correction for actions that are viewed as outside of expected norms. This is supported by studies showing that, at the overall classroom level, students’ academic engagement, social competence, and emotional adaptation are associated with the level of warmth and emotional sensitivity that teachers demonstrate to the class (for a review, see Troop‐Gordon 2015). Teachers also develop distinct relationships with individual students. Compared to classmates who do not present problem behavior, school-age students with behavior problems in the classroom are more likely to have strained or conflictual relationships with teachers (e.g., Baker 2006, O’Connor et al. 2011). Similarly, early adolescents reporting lower levels of teacher support and higher levels of stress in their relationships with teachers report lower levels of socio-emotional adjustment and higher levels of involvement in bullying, both as bullies and as victims (e.g., Demol et al. 2021, Huang et al. 2018, Murray-Harvey & Slee 2010). Collectively, these studies emphasize the role of teachers as significant figures in the school setting, who may be able to moderate microaggressions against school-age children of sexual minority parents and promote a classroom environment that is sensitive to family diversity.

With respect to the study context, two recent Italian studies identified widespread negative beliefs about—and perceptions of—child development in sexual minority parent families among teachers and educators working in nursery schools, kindergartens, and primary schools (Baiocco et al. 2020, De Simone et al. 2020). This result takes on particular relevance when viewed through the lens of developmental intergroup theory (Bigler & Liben 2006), as, during the elementary school years, children may develop prejudicial beliefs and attitudes towards sexual minority parent families because the gender composition of the parents is perceptually salient, and sexual minority parent families are less common than heterosexual parent families.

However, children of sexual minority parents may be less perceptually discriminable than their parents (Farr et al. 2019). As developmental intergroup theory would predict, prejudice towards groups that are less perceptually salient can still develop, in line with the sociocultural environment (e.g., media attention and public debate surrounding issues related to diverse family forms) and adults’ (even unintentional) behavior modeling. These contextual factors may significantly contribute to shaping the school experiences of children born to sexual minority parents through assisted reproduction in Italy, as teachers may (voluntarily or not) endorse heterosexism when celebrating only “Mother’s Day” and “Father’s Day,” instead of the more inclusive “Parents’ Day”; or when endorsing the cultural value of bionormativity, by conveying that children should look like both of their parents.

Given the subtle and daily nature of many microaggressions in the school setting, teachers are often unaware of their occurrence (Troop‐Gordon 2015). Under these circumstances, when parents are not available, it is crucial that microaggressed children perceive warm relationships with their teachers and consider them reliable adults to whom they can turn when upset or worried. This evokes an attachment perspective on the child–teacher relationship, stressing the importance of the affective quality of the dyad (Pianta 1992, Verschueren 2015). A number of conceptual models have been used to describe child–teacher relationships—including socio-motivational, socialization, and interpersonal theories, as well as social support models (Pianta & Allen 2008). Each of these emphasizes the importance of students’ perceptions of emotional support or relatedness. Uniquely, the attachment framework adds a developmental perspective, as it posits that students bring their early relational models of social relationships and the social world to the classroom. These internal working models inevitably guide students’ understanding of relationships (with, e.g., teachers) and influence the quality of their relationships by shaping their interpretations of their interactions (with, e.g., teachers) (Bowlby 1988).

Although most school-age children’s bonds with their teachers are not as significant attachment bonds as those established with their parents, teachers may still be regarded as temporary or ad hoc attachment figures, to the extent that they provide a secure base for children to explore their learning and social environments and a safe haven to turn to in times of stress (Bowlby 1988, Verschueren 2015). From this perspective, when a child is vulnerable to peer microaggressions at school—as might be the case for children of sexual minority parents—their attachment bond to a teacher is key, as their attachment system is likely to be activated more readily and their capacity for self-regulation may be limited by their relatively young age. In this vein, some studies have shown that child–teacher relationships characterized by closeness, warmth, and support are associated with better social skills and academic performance, as well as with fewer externalizing problems in children (Koenen et al. 2019, Pianta & Stuhlman 2004). Conversely, child–teacher relationships that are poor, problematic, or conflictual represent a further risk factor in the context of adversity (e.g., peer microaggressions) (Berry & O’Connor 2010, Howes et al. 1994, Rasheed et al. 2020).

Finally, from a developmental perspective, Bowlby considered development a dynamic process in which “established patterns of adaptation may be transformed by new experiences while, at the same time, new experiences are framed by, interpreted within, and even in part created by prior history of adaptation” (Sroufe 2005, p. 350). Scaffolding on this idea, child–teacher relationships could potentially compensate for children’s previous attachment experiences and serve to regulate children’s social and emotional adjustment. For this reason, child–teacher relationships are increasingly viewed as key developmental contexts—not only for children’s academic progress, but also for their socio-emotional trajectories (Pianta & Stuhlman 2004). However, although researchers have increasingly interpreted child–teacher relationships as a context for child socio-emotional development within an attachment framework, to date, such research has not been applied to examine the role of child–teacher relationships in reducing or amplifying the negative impact of peer microaggressions on children’s social skills in the school setting.

## Current Study

The impact of peer microaggressions and the child–teacher relationship on the social skills of children with sexual minority parents through assisted reproduction still remains to be explored. The current study uses a mixed-method, multi-informant, two-wave longitudinal design to examine the moderating role of child–teacher relationships in the association between child-reported peer microaggressions and children’s social skills (as rated by parents and teachers after 18 months) among school-age children of lesbian mothers through donor insemination and gay fathers through surrogacy in Italy. Specifically, it is hypothesized that: (a) higher child–teacher relationship quality will buffer against the detrimental effect of peer microaggressions on social skills; while (2) lower child–teacher relationship quality will represent a further risk factor for child social skills in the context of more intense peer microaggressions. In addition, for descriptive reasons, the study conducts a preliminary investigation of the frequency and intensity of peer microaggressions experienced by children of lesbian and gay parents, hypothesizing that most children will report at least one experience of microaggression and describe microaggression experiences that are of medium to high intensity, on average, in line with the conservative context in which children are socialized in Italy. In this vein, it is expected that these children will report that peers have addressed them using insensitive phrases and terms—or even derogated them through name-calling—though perhaps inadvertently and without grasping the profound significance of their words.

## Method

### Participants

Data were gathered from the first (W1) and second (W2) waves of a longitudinal study of sexual minority parent families with children in middle childhood (see Carone et al. 2020b, Carone et al. 2020c). Participants were 37 lesbian mother families formed through donor insemination (*n* = 74 parents and 37 children) and 33 gay father families formed through surrogacy (*n* = 66 parents and 33 children), all with a child aged 6–12 years at W1 and 7.6–13.6 years at W2, and residing in Italy. In each family, the teacher who spent the most time with the child in the classroom was also involved. Of the 70 teachers contacted, 55 agreed to participate (78.6% response rate). All teachers were women, with a mean age of 44.26 years (*SD* = 5.28). At W1, 27 lesbian mother families and 24 gay father families were recruited in the context of a larger, in-depth study of child adjustment and parenting in gay father surrogacy families (Carone et al. 2018). To increase the sample size, a further 10 lesbian mother families and 9 gay father families with children in the same age range were recruited. Specifically, multiple strategies were used to recruit as diverse a sample as possible, through the main Italian association of same-sex parents (*n* = 25, 35.7%), same-sex parent web groups and forums (*n* = 22, 31.4%), events at which same-sex parents were in attendance (*n* = 9, 12.9%), and snowballing (*n* = 14, 20.0%). The inclusion criteria for both lesbian mother families and gay father families were that the couple had lived together since the child’s birth, resided in Italy, and had conceived through donor insemination and surrogacy, respectively. Table 1 presents the sociodemographic information for each group.

**Table 1:** Sociodemographic information, by family type (*n* = 70)

	Lesbian mother families (*N* = 37)	Gay father families (*N* = 33)			
*Family variables*	*n* (%)	*n* (%)	*Χ*^*2*^(df)	*p*	
Child gender			0.064(1)	0.800	
Boy	19 (51.3)	15 (45.6)			
Girl	18 (48.7)	18 (54.5)			
Number of siblings at W2			0.368(2)	0.832	
0	10 (27.0)	10 (30.3)			
1	22 (59.5)	20 (60.6)			
2 or more	5 (13.5)	3 (9.1)			
Length of parents’ relationship at W2			0.892(2)	0.640	
<10 years	7 (18.9)	7 (21.2)			
11–15 years	14 (37.8)	9 (27.3)			
>15 years	16 (43.2)	17 (51.5)			
Marital status at W2			1.080(2)	0.583	
Civil partnership in Italy	23 (62.2)	20 (60.6)			
Only married/civil partnership abroad	12 (32.4)	9 (27.3)			
Unmarried/no civil partnership	2 (5.4)	4 (12.1)			
Residence			1.226(2)	0.542	
Northern Italy	11 (29.7)	14 (42.4)			
Central Italy	22 (59.5)	16 (48.5)			
Southern Italy	4 (10.8)	3 (9.1)			
	*M* (*SD*)	*M* (*SD*)	*F*(*df*)	*p*	*Ŋ*^*2*^_*p*_
Child age at W1 (months)	99.27 (18.49)	99.39 (20.85)	<0.01(1,68)	0.979	<0.001
Child age at W2 (months)	117.49 (18.68)	117.73 (20.93)	<0.01 (1,68)	0.960	<0.001
Household income at W1 (euros)	70,540.54 (28,541.73)	123,681.82 (67,014.90)	19.36(1,68)	<0.001	0.222
Household income at W2 (euros)	79,243.24 (28,522.80)	114,772.73 (30,197.03)	25.61(1,68)	<0.001	0.274
Individual variables	*n* (%)	*n* (%)	*Χ*^*2*^(df)	*p*	
Parent ethnicity (White)^a^	69 (93.2)	60 (90.9)	0.263 (1)	0.608	
Parent educational level (bachelor’s degree or higher)	52 (70.3)	51 (77.2)	0.880 (1)	0.348	
Parent occupation at W2 (professional/managerial)	49 (66.2)	52 (78.8)	2.744 (1)	0.098	
Parent work status at W2 (full-time)	59 (79.7)	66 (100.0)	14.984 (1)	<0.001	
	*M* (*SD*)	*M* (*SD*)	*F*(*df*)	*p*	*Ŋ*^*2*^_*p*_
Parent age at W1 (years)	41.68 (4.74)	47.05 (6.14)	10.50 (1,68)	0.002	0.204
Parent age at W2 (years)	42.22 (5.82)	48.85 (6.80)	27.86 1,68)	<0.001	0.168

*W* Wave. ^a^The remaining parents self-identified their ethnicity as Hispanic. Chi-square tests were reported with Yates’s correction for continuity. Percentages may not equal 100, due to rounding.

### Procedure

The research protocol was approved by the Ethics Committees of the Department of Developmental and Social Psychology, Sapienza University of Rome (at W1), and the Department of Brain and Behavioral Sciences, University of Pavia (at W2). Three researchers at W1 and one researcher at W2 assessed families at home. At W1, parents gave consent for their children to participate and to be contacted approximately 18 months later for follow-up; children also provided verbal assent. Each child was reminded that their responses would be confidential and that they could terminate their participation in all or part of the study at any time; such information was conveyed to children in an age-appropriate manner, both prior to and during their participation.

Parents were asked to pass an information sheet about the study to the teacher who spent the most time with their child in the classroom. The information sheet contained the researchers’ contact details, in the event that teachers wanted more information about the study before deciding whether to participate. Parents were informed that they were not obliged to pass the information on, and teachers were informed that their responses would not be reported back to the child’s family or the school. The first author sent teachers who agreed to take part a scanned copy of the questionnaires, by email. Approximately 18 months later (W2), parents were contacted again to participate in the study, and all agreed to take part. Again, the teacher who spent the most time with the target child in the classroom was asked to participate in the study. Among the 55 families (i.e., 28 lesbian mother families, 27 gay father families) for which teachers participated at both waves, 11 families (i.e., 6 lesbian mother families, 5 gay father families) involved different teachers at W1 and W2, as the children transitioned from elementary to secondary school.

### Measures

#### Peer microaggressions (at W1; interviewer ratings)

At W1, children participated in a semi-structured interview that was designed to uncover any experiences of teasing and bullying by peers due to having sexual minority parents and/or being born through assisted reproduction (Farr et al. 2016a, Vanfraussen et al. 2002). Two of the three researchers involved in W1 with expertize in child development and family diversity conducted the interviews, which lasted 20–30 min, on average. Both interviewers were non-parent individuals (i.e., a cisgender heterosexual woman and a cisgender gay man) who had received formal training on interview techniques with school-age children. Interviews were conducted with children alone (without the parents’ presence), while their parents completed the questionnaires in another room of the family home. This arrangement aimed at protecting children’s privacy and preventing any (involuntary) parental influence. Children were asked the following question: “At school, have you ever been made fun of or teased (e.g., with words, called names, had mean things said to you) by your schoolmates due to having two moms/two dads and/or being born through assisted reproduction?” If children responded “Yes,” their experiences were probed further via the following questions: “Can you describe what happened?”; “How many times in the last year?”; “Why did your schoolmates made fun of or tease you?”; “What did they say?”; “How did you feel?”; and “What did you do when it happened?”.

Following the coding manual developed by Farr et al. (2016a), two undergraduate students globally coded children’s entire interview transcripts, using deductive thematic analysis—a process that allows patterns (i.e., themes) within the data to be identified, analyzed, and reported (Braun & Clarke 2006). The coding process was theory-driven (i.e., microaggression framework; Braun & Clarke 2021). Deductive thematic analysis was chosen over other qualitative methods (e.g., qualitative content analysis) because it explicitly involves theoretical assumptions (i.e., about microaggressions) and enhances dependability, such that the systematic process can be enacted across a relatively large data set (Braun & Clarke 2021, Patton 2002) while maintaining theoretical flexibility. Because no prior research had studied the microaggression experiences of children of sexual minority parents through assisted reproduction, it was important that the deductive thematic analysis allow participants’ voices and experiences to be heard while also considering the context of the peer microaggressions. The coding process included seven stages, adapted from Braun and Clarke’s (2006) structured thematic analysis framework.

During the first stage of data analysis, two coders immersed themselves in the data by reading approximately 30% (*n* = 20) of the interview transcripts to gain an understanding of the context of each child and their experiences (Phase 1—*Become familiar with the data*). The first author systematically chose these initial transcripts, aiming at an equal representation of female and male respondents from both lesbian mothers and gay fathers, and of varying ages (i.e., 6–12 years). The coders were only aware of children’s family type. Collaboratively, the coders established a comprehensive and thorough list of themes that appeared in the data and defined a *microaggression* unit as the most basic segment of the raw data that could be assessed in a meaningful way regarding the topic under investigation (Phase 2—*Set up and unitize the data*).

After identifying and comparing microaggressions in the data, the coders clustered these microaggressions into potential themes (i.e., *heterosexism*, *teasing and bullying*, *negative stereotypes and overt discrimination*, *public outing*, *questioning legitimacy of family/bionormativity*, *spokesperson*; Farr et al. 2016a), generating a provisional typology that was refined in subsequent steps. The first author supervised this phase, ensuring that both coders were coding the same data, while also helping to code, interpret, and thematically classify microaggressions. Once all potential themes were identified, those with substantial overlap were merged. Microaggressions that did not appear to fit with any theme or were not included in Farr et al. 2016a codebook were placed in an *other* category (e.g., *alternative path to parenthood would have been better*, “Why didn’t your dads adopt you instead of using surrogacy?”; *phantom birth other*, “Don’t you know your donor? That is because he doesn’t want to be involved with you, he doesn’t care about you”; *commerce in assisted reproduction*, “My mother told me that surrogacy is like renting a womb and that these parents pay to have children. Are you that kind of child?”). Microaggressions were reported when children provided distinct responses (e.g., a few words or several sentences) that reflected any of the themes identified in the coding manual, including the *other* category. After individual ratings were assigned, the two coders met to determine the final codes, through consensus. During discussions, the first author posed as the moderator, taking detailed notes about any necessary changes to the codebook and rating system, and making executive decisions in the event of disagreement. Following this initial review, a template was created as a guide for coding the interview transcripts. The template was subsequently reviewed by the first author and amended according to his notes (Phase 3—*Create and finalize themes in the codebook*).

Before engaging in the coding process, both coders read general articles about microaggressions, children’s experiences in sexual minority parent families, and families formed through assisted reproduction (e.g., Bos & Gartrell 2020, Carone et al. 2018, Farr et al. 2016a, Garber & Grotevant 2015, Golombok 2020). They also completed 20 h of training with the first author on the interview codes and rating anchor points, using 20 transcripts collected by the research team for another project on diverse family forms (Phase 4—*Train coders*). Once themes were identified and finalized in the codebook, each coder independently re-read the initial 30% of interview transcripts (*n* = 20) and refined their previous coding of microaggressions. During this process, the coders and the first author met weekly to ensure that each identified microaggression reflected only one microaggression. The team discussed any inconsistences until agreement was reached. Each transcript had a corresponding document containing all instances of unitized microaggressions and their intensities (phase 5—*Unitize codes with coding team*). Following this consensus process, one coder coded all of the remaining interviews (*n* = 50), while the second coder coded only 15 further interviews (Phase 6—*Code units with coding team using codebook*).

Finally, once all 70 interviews were coded for microaggressions, each of the two coders assigned an intensity level (i.e., low, medium, or high) to each microaggression, in accordance with the coding manual (Farr et al. 2016a) (Phase 7—*Produce the report*). A score of 0 was assigned when the child did not report any microaggression. A rating of “low” (i.e., 1) corresponded to microinvalidations (i.e., behaviors that subtly singled out or minimized the child; e.g., “They ask me a lot of questions about where my mom is” —*heterosexism* theme); a rating of “medium” (i.e., 2) corresponded to microinsults (i.e., expressions that conveyed insensitivity and demeaned the child; e.g., hearing “You’re gay” among peer groups as a derogatory phrase—*negative stereotypes and overt discrimination* theme); and a rating of “high” (i.e., 3) corresponded to microassaults (i.e., intentionally insulting behaviors such as name-calling that derogated the child; e.g., “I was told I was going to hell because I had two moms”—*teasing and bullying* theme) (Sue et al. 2007). Interrater reliability was calculated on the double-coded interviews (50% of the entire set, *n* = 35) for the identification of themes and intensity levels, and was excellent, with intraclass correlation coefficients (ICCs) ≥ 0.83 for each variable. When children reported multiple microaggressions, these were coded and rated separately; subsequently, for each child, a single peer microaggression score was calculated by dividing the sum of the intensity of each microaggression by the number of microaggressions reported. Thus, the peer microaggression score reflected both the intensity and the frequency of children’s microaggressions.

#### Child–teacher relationship quality (at W1; child ratings)

Attachment-related dimensions in the child–teacher relationship, as perceived by children, were measured at W1 using the two attachment scales of the Network of Relationships Inventory—Behavioral Systems Version Questionnaire (NRI-BSV; Furman & Buhrmester 2009): *Seek Safe Haven*, which measures the extent to which respondents rely on their relational partner as a safe haven when upset or distressed (example items: “How much do you seek out your teacher when you’re upset?”; “How much do you turn to your teacher for comfort and support when you are troubled about something?); and *Seek Secure Base*, which measures the extent to which respondents use their relational partner as a secure base from which to engage in non-attachment behaviors (example items: “How much does your teacher encourage you to try new things that you’d like to do but are nervous about?”; “How much does your teacher show support for your activities?). In addition, the *Negative Interaction* subscale was included to obtain a measure of negative relationship quality (example items: “How much do you and your teacher argue with each other?”; “How much do you and your teacher criticize each other?”) (De Laet et al. 2014). The two attachment subscales are comprised of three items, whereas the Negative Interaction subscale is a nine-item index of the degree to which respondents experience conflict, antagonism, and criticism in their relationship. Thus, in the current study, the final scale was comprised of 15 items. Children rated items with reference to the teacher with whom they spent the most time in the classroom, using a 5-point Likert-type scale ranging from 1 (*little or none*) to 5 (*the most*). Scores for each scale were computed by averaging the item scores, with reversed scores used for the Negative Interaction subscale. Higher scores indicated a more secure child–teacher relationship. The NRI-BSV is a validated and standardized questionnaire used to assess child–teacher relationship quality with children in middle childhood and early adolescence from an attachment perspective, showing acceptable-to-good reliability (de Laet et al. 2014, Furman & Buhrmester 2009). In the current study, all children completed the questionnaire using pencil on paper. One of the researchers assisted them in the event that they expressed doubts or questions about item wording. Each item was also preliminary read aloud to the youngest children (aged 6–7 years), to ensure they understood the questions correctly. To the best of our knowledge, this study was the first to use the NRI-BSV with children of sexual minority parents through assisted reproduction. Cronbach’s alpha was 0.79.

#### Child social skills (at W1 and W2; parent and teacher ratings)

At both waves, in each family, both parents and the teacher who spent the most time with the child in the classroom completed the Social Skills Rating System (SSRS; Gresham & Elliott 1990) to evaluate children’s social behavior, at home and at school, respectively. The SSRS teacher version (SSRS-T) is a 30-item measure of children’s social behavior, consisting of three subscales with 10 items each, rated on a 3-point frequency scale ranging from 0 (*never*) to 2 (*very often*). The *Cooperation* subscale assesses behaviors such as helping others, sharing materials, and complying with rules and directions (example item: “Follows your directions”) The *Assertion* subscale assesses initiating behaviors, such as asking others for information, introducing oneself, and responding to the actions of others (example item: “Invites others to join in activities”). The *Self-Control* subscale measures behaviors that emerge in both conflict (e.g., responding appropriately to teasing) and non-conflict situations (e.g., taking turns and compromising) (example item: “Responds appropriately when pushed or hit by other children”). From these subscales, a total social skills score can be computed, ranging from 0–60. Conversely, the SSRS parent version (SSRS-P) consists of four subscales of 10 items each. In addition to the Cooperation, Assertion, and Self-Control subscales described above, the SSRS-P also includes a *Responsibility* subscale, which measures behaviors demonstrating the child’s ability to communicate with adults and their concern for property or work (example item: “Requests permission before leaving the house”) However, as two items load onto two factors, the total scale consists of 38 items, with a score range of 0–80. Both the SSRS-P and the SSRS-T have demonstrated excellent psychometric properties in terms of internal consistency and test–retest reliability, relationship to other measures, and factor structures (see Gresham & Elliott 1990). In the current study, Cronbach’s alphas were 0.88 and 0.85 for the parent and teacher versions, respectively.

### Analytic Strategy

All analyses were conducted using the R software (R Core Team 2021). The only missing data pertained to 15 teacher evaluations of child social skills at W1, which were subsequently not collected at W2. As it was not possible to handle missing data, all analyses using teacher ratings of child social skills pertained to only 55 families (i.e., 28 lesbian mother families, 27 gay father families), instead of the full set of 70. As a preliminary analysis, the non-parametric Mann-Whitney U test was performed to check whether, within these 55 families, there were differences in teacher scores based on whether the same teacher evaluated children’s social skills at W1 and W2. Furthermore, for the entire sample, the analyses calculated means, standard deviations, and frequencies by family type and child gender. Potential differences in microaggressions, child–teacher relationship quality, and child social skills across family type and child gender were preliminarily tested. Given the dramatic age range of the sample, child age was entered as a covariate. Finally, bivariate correlations were used to identify any associations among the sociodemographic and study variables.

Next, to assess whether microaggressions led to a decline in children’s social skills over time, moderated by child–teacher relationship quality, a mixed model regression with W2 values as the outcome and W1 scores as the predictor was run to calculate the residualized change score of social skills. The standardized residual values were then saved and used in later analyses. This conservative approach allowed for the potential influence of baseline social skills on change in social skills at W2 to be controlled. Subsequently, multiple linear regression (MLR) and hierarchical linear modeling (HLM) were performed using teachers’ (i.e., one change score per child) and parents’ (i.e., two change scores for the same child) social skills ratings, respectively.

HLM was used for the theoretical and statistical evidence: since there were 140 parents nested in 70 families, it was necessary to control for within-family correlations in the outcome scores to provide more accurate standard errors and associated hypothesis tests (Smith et al., 2020). Also, the intra-class correlations for the unconditional model predicting children’s social skills at W2 was 67%, higher than the 25% cutoff suggested for the use of HLM (Guo, 2005). For both MLR and HLM, continuous predictor variables were grand mean centered and dichotomous variables were effects coded (i.e., family type: gay father family = −1, lesbian mother family = 1; child gender: male = −1, female = 1). Given the relatively small sample size, the aim was to generate enough statistical power whilst still accounting for the sociodemographic differences between family types. Therefore, sociodemographic variables that differed between groups—along with family type, child age, and gender—were first tested alone, then retained in the final model only if they approached statistical significance (*p* < 0.05).

The Johnson–Neyman technique (Preacher et al. 2006) was performed to evaluate the interactive effects of microaggressions and child–teacher relationship quality through an inspection of the range of values (i.e., regions of significance) of the moderator (child–teacher relationship quality) for which the predictor (microaggressions) and outcome (social skills) were significantly associated. This technique was selected over simple slopes analysis because it uses regions of significance to highlight all possible values of the moderator for which there are significant regressions of the outcome on the predictor, instead of probing only two arbitrarily specified levels (i.e., child–teacher relationship quality values that are 1 SD above and below the mean, even though it is a continuous dimension without a natural break point; for a wider discussion, see Dearing & Hamilton 2006).

## Results

### Descriptives and Associations among the Study Variables

In both family types, approximately two-thirds of children reported at least one peer microaggression; conversely to expectations, among the entire sample, peer microaggressions were, on average, of low intensity. Detailed statistics are reported in the tables: specifically, Table 2 displays the bivariate correlations among the study variables, whereas Table 3 describes peer microaggressions, child–teacher relationship quality, and child social skills, by family type and child gender.

**Table 2:** Associations among the sociodemographic variables at W2; microaggressions and child–teacher relationship at W1; and social skills at W1 and W2 (*N* = 140 parents, 70 children, 55 teachers)

Variables	1.	2.	3.	4.	5.	6.	7.	8.	9.	10.
1. Child age at W2	1									
2. Parent age at W2	0.32^***^	1								
3. Number of siblings at W2	0.19	0.07	1							
4. Household income at W2	0.16^†^	0.26^**^	0.04	1						
5. Microaggressions at W1	0.33^***^	0.16^†^	0.09	0.02	1					
6. Child–teacher relationship at W1	0.28^*^	0.18^*^	0.06	0.05	0.04	1				
7. Social skills-p at W1	0.12	0.15^†^	<0.01	0.14	<0.01	0.13	1			
8. Social skills-t at W1	0.12	0.12	−0.08	0.12	−0.01	0.14	0.69^***^	1		
9. Social skills-p at W2	0.25^**^	0.19^*^	0.07	0.18^*^	−0.16^†^	0.46^***^	0.47^***^	0.44^***^	1	
10. Social skills-t at W2	−0.09	0.08	−0.02	0.18^†^	−0.33^*^	0.38^**^	0.26^**^	0.40^**^	0.62^***^	1
*M*	117.60	45.87	0.83	95,992.86	1.27	48.76	58.41	43.45	60.65	45.22
*SD*	19.63	5.37	0.63	35,153.59	1.05	11.91	7.96	6.53	10.85	11.83

-p = parent ratings. -t = teacher ratings.

^†^*p* < 0.10. ^*^p < 0.05. ^**^p < 0.01. ^***^p < 0.001.

**Table 3:** Means and standard deviations of peer microaggressions and child–teacher relationship quality at W1, and social skills at W1 and W2, by family type and child gender (*N* = 70 families)

	Lesbian mother families			Gay father families		
	Total (*N* = 37)	Male children (*n* = 19)	Female children (*n* = 18)	Total (*N* = 33)	Male children (*n* = 15)	Female children (*n* = 18)
	*N (%)*	*n (%)*	*n (%)*	*N (%)*	*n (%)*	*n (%)*
Peer microaggressions frequency at W1						
No microaggressions	11 (29.7)	4 (21.1)	7 (38.9)	11 (33.3)	6 (40.0)	5 (27.8)
Yes microaggressions	26 (70.3)	15 (78.9)	11 (61.1)	22 (66.7)	9 (60.0)	13 (72.2)
	*M (SD)*	*M (SD)*	*M (SD)*	*M (SD)*	*M (SD)*	*M (SD)*
Peer microaggressions score at W1^a^	1.30 (1.04)	1.48 (1.05)	1.10 (1.03)	1.24 (1.08)	1.20 (1.15)	1.27 (1.05)
Child–teacher relationship quality at W1	50.24 (11.71)	50.58 (13.49)	49.89 (9.87)	47.09 (12.08)	45.93 (11.84)	48.06 (12.54)
Social skills at W1 (parent ratings)	59.15 (11.08)	59.45 (9.49)	55.17 (6.65)	62.33 (10.50)	59.93 (8.22)	59.28 (6.84)
Social skills at W1 (teacher ratings)	42.86 (6.72)	45.07 (7.42)	40.64 (5.30)	44.07 (6.40)	45.50 (6.24)	43.24 (6.53)
Social skills at W2 (parent ratings)	59.15 (11.08)	60.66 (11.81)	57.56 (10.35)	62.33 (10.50)	63.10 (11.91)	61.69 (9.48)
Social skills at W2 (teacher ratings)	44.86 (11.15)	46.43 (10.32)	43.29 (12.10)	45.59 (12.71)	44.60 (12.38)	46.18 (13.24)

^a^For each child, a single peer microaggression score was calculated by dividing the sum of the intensity of each microaggression by the number of microaggressions reported. Thus, the peer microaggression score reflected both the intensity and the frequency of children’s microaggressions. Percentages may not equal 100 due to rounding. For teacher-rated social competencies at W2, *N* = 55.

### Preliminary Differences in Teacher Ratings of Child Social Skills

Among the 55 families for which teacher ratings of child social skills were available at both waves, the Mann-Whitney U test did not indicate any group differences between those in which different teachers participated across the two waves (mean rank: 26.82) and those in which the same teacher participated at both waves (mean rank: 26.82), U = 229.00, *p* = 0.784.

### Preliminary Differences across Family Type and Child Gender

Four analyses of covariance (ANCOVAs) were run to examine potential differences in peer microaggressions and child–teacher relationship quality at W1, and teacher ratings of social skills at W1 and W2, by family type and child gender, controlling for child age at W1. Regarding peer microaggressions at W1, there were no differences between lesbian and gay parent families, *F*(1,65) = 0.07, *p* = 0.799, ŋ^2^_p_ = 0.001, or male and female children, *F*(1,65) = 0.36, *p* = 0.551, ŋ^2^_p_ = 0.005; neither was there a significant interaction between family type and child gender, *F*(1,65) = 0.97, *p* = 0.328, ŋ^2^_p_ = 0.015. Conversely, child age was a significant covariate, *F*(1,65) = 5.18, *p* = 0.026, ŋ^2^_p_ = 0.074. Likewise, child–teacher relationship quality at W1 was similar between gay and lesbian parent families, *F*(1,65) = 1.40, *p* = 0.242, ŋ^2^_p_ = 0.021, as well as between male and female children, *F*(1,65) = 0.08, *p* = 0.781, ŋ^2^_p_ = 0.001; the interaction between family type and child gender was not significant, *F*(1,65) = 0.36, *p* = 0.554, ŋ^2^_p_ = 0.005, whereas child age was a significant covariate, *F*(1,65) = 5.88, *p* = 0.018, ŋ^2^_p_ = 0.083.

Also, teacher ratings of social skills at W1 revealed no differences between children of lesbian versus gay parents, *F*(1,50) = 0.73, *p* = 0.396, ŋ^2^_p_ = 0.014, or female versus male children, *F*(1,50) = 3.20, *p* = 0.080, ŋ^2^_p_ = 0.060. Neither the interaction between family type and child gender, *F*(1,50) = 0.45, *p* = 0.508, ŋ^2^_p_ = 0.009, nor the influence of child age, *F*(1,50) = 0.62, *p* = 0.434, ŋ^2^_p_ = 0.012, was significant. Finally, at W2, teachers reported similar social skills between children of lesbian versus gay parents, *F*(1,50) = 0.43, *p* = 0.514, ŋ^2^_p_ = 0.009; as well as between female and male children, *F*(1,50) = 0.09, *p* = 0.764, ŋ^2^_p_ = 0.002. Also, neither the interaction between family type and child gender, *F*(1,50) = 0.43, *p* = 0.514, ŋ^2^_p_ = 0.009, nor the influence of child age, *F*(1,50) = 0.40, *p* = 0.528, ŋ^2^_p_ = 0.008, was significant.

When parent ratings of social skills at W1 were applied, the linear mixed model indicated no differences across family types, *estimate* = −0.03, *SE* = 0.24, *p* = 0.896, or child gender, *estimate* = −0.05, *SE* = 0.25, *p* = 0.851. Furthermore, neither the interaction between family type and child gender, *estimate* = −0.34, *SE* = 0.34, *p* = 0.321, nor the influence of child age, *estimate* = 0.12, *SE* = 0.08, *p* = 0.156, was significant. Likewise, at W2, parents reported no differences in child social skills across family types, *estimate* = −0.18, *SE* = 0.31, *p* = 0.554, or child gender, *estimate* = −0.10, *SE* = 0.31, *p* = 0.762. The interaction between family type and child gender was not significant, *estimate* = −0.18, *SE* = 0.43, *p* = 0.672; whereas child age was a significant covariate, *estimate* = 0.25, *SE* = 0.11, *p* = 0.021.

### Longitudinal Influence of Microaggressions on Social Skills, Moderated by Child–Teacher Relationship Quality

Several HLMs were computed and compared using fit indices to examine the moderating role of child–teacher relationship quality at W1 in the longitudinal influence of microaggressions at W1 on social skills at W2 (as rated by parents). For the sake of concision, the following only presents the model that best fit the data. Regarding sociodemographic variables, to preserve statistical power, family type and child gender were excluded from the analyses given their non-significant effect on any of the study variables, as examined above.

Table 4 reports all of the performed models. First, a null model with no predictor was run (model 0); next, child age at W2 was tested as the main predictor (model 1); subsequently, microaggressions and child–teacher relationship quality at W1 were included as additive terms (model 2); finally, the interaction between microaggressions and child–teacher relationship quality at W1 (model 3) was introduced. Following the convention that the model with the highest global variance (see TCD; Bollen, 1989) and the lowest BIC (Schwarz, 1978) should be considered the best explanation of the data, model 3 resulted as the best model and is therefore explained in detail below. In this model, parents reported fewer social skills at W2 in children who reported more intense microaggressions, *estimate* = −0.18, *SE* = 0.07, *p* = 0.017, and a lower child–teacher relationship quality at W1, *estimate* = 0.25, *SE* = 0.08, *p* = 0.002. Also, the interaction between microaggression intensity and child–teacher relationship quality was significant, *estimate* = 0.45, *SE* = 0.07, *p* < 0.001. Finally, social skills increased with child age, *estimate* = 0.22, *SE* = 0.08, *p* = 0.011.

**Table 4:** Longitudinal influence of microaggressions on parent-rated social skills, moderated by child–teacher relationship quality and model fit indices (*N* = 140 parents and 70 children)

	Outcome: Change in social skills-p at W2					
Predictors	estimate *(SE)*	CI [2.5%, 97.5%]	*p*	TCD_*marginal*_	TCD_*conditional*_	BIC
Model 0 (null model)				0.00	0.59	384.30
Model 1				0.05	0.05	415.15
Fixed effects						
Intercept	<0.01 (0.08)	−0.16, 0.16	1.000			
Child age	0.22 (0.08)	0.06, 0.39	0.008			
Random effects	*SD*	*Variance ICC*	*p*			
Intercept (within-couple variance)	0.00	0.00 0.00	1.000			
Residual	0.98	0.96				
Model 2				0.28	0.60	377.69
Fixed effects						
Intercept	<0.01 (0.09)	−0.17, 0.17	1.000			
Child age	0.17 (0.10)	−0.01, 0.36	0.070			
Microaggressions at W1	−0.27 (0.09)	−0.45, −0.09	0.004			
Child–teacher relationship quality at W1	0.43 (0.09)	0.25, 0.61	<0.001			
Random effects	*SD*	*Variance ICC*	*p*			
Intercept (within-couple variance)	0.58	0.33 0.45	<0.001			
Residual	0.64	0.41				
Model 3				0.46	0.60	355.59
Fixed effects						
Intercept	−0.05 (0.07)	−0.19, 0.10	0.501			
Child age	0.22 (0.08)	0.07, 0.37	0.011			
Microaggressions at W1	−0.18 (0.07)	−0.33, −0.04	0.017			
Child–teacher relationship quality at W1	0.25 (0.08)	0.10, 0.41	0.002			
Microaggressions at W1 * Child–teacher relationship quality at W1	0.45 (0.07)	0.31, 0.59	<0.001			
Random effects	*SD*	*Variance ICC*	*p*			
Intercept (within-couple variance)	0.37	0.14 0.25	0.035			
Residual	0.64	0.41				

Model 3 best fit the data, with both highest TCD and lowest BIC. *CI* Confidence interval; *BIC* Bayesian information criterion (Schwarz, 1978); *TCD* Total coefficient determination. TCD_*marginal*_ represents the proportion of the total variance explained by the fixed effects, whereas TCD_*conditional*_ represents the proportion of the variance explained by both fixed and random effects.

**Fig. 1:**
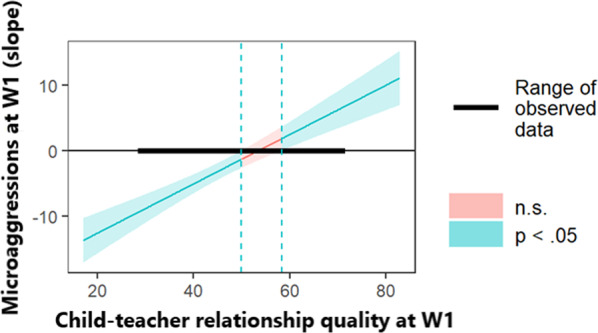
Johnson-Neyman plot using parents' reports of child social skills.

Subsequently, the Johnson–Neyman technique was used to identify the child–teacher relationship quality regions of significance in which the effect of microaggression intensity on social skills was significant. The findings indicated that when the child–teacher relationship quality score was outside of the interval 50.02–58.34, microaggressions significantly predicted social skills at *p* < 0.05 (see Fig. 1). Given that the range of observed child–teacher relationship quality values was 29.00–71.00, two patterns of interaction were significant: for 54.29% of the children, more intense microaggressions at W1 predicted lower social skills at W2 when they reported a lower W1 child–teacher relationship quality; whereas for 27.14% of the children, more intense microaggressions at W1 predicted greater social skills at W2 when they reported a higher W1 child–teacher relationship quality.

**Fig. 2:**
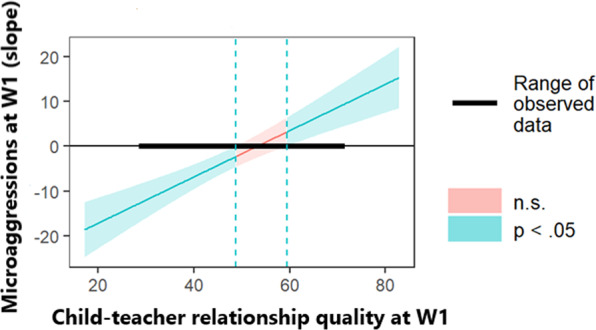
Johnson-Neyman plot using teachers' reports of child social skills.

When teacher ratings of child social skills at W2 were input into several linear regression models, similar results were found—with the exception that child age lost significance, *β* = −0.16, *SE* = 0.09, *p* = 0.082. Specifically, fewer social skills at W2 were predicted by more intense W1 microaggressions, *β* = −0.25, *SE* = 0.09, *p* = 0.010, and low W1 child–teacher relationship quality, *β* = 0.35, *SE* = 0.09, *p* < 0.001. Also, the interaction between microaggressions and child–teacher relationship quality was significant, *β* = 0.59, *SE* = 0.09, *p* < 0.001. The follow-up Johnson-Neyman technique indicated that, when the child–teacher relationship quality score was outside of the interval 48.79–59.41, microaggressions were significant predictors of social skills at *p* < 0.05 (see Fig. 2). That is, for 50.91% of the children, more intenseW1 microaggressions significantly predicted lower W2 social skills when they reported a lower W1 child–teacher relationship quality; whereas for 14.55% of the children, more intense W1 microaggressions predicted greater W2 social skills when they reported a higher child–teacher relationship quality. Table 5 reports the full statistics and model fit indices.

**Table 5:** Longitudinal influence of microaggressions on teacher-rated social skills, moderated by child–teacher relationship quality and model fit indices (*N* = 55 teachers and 55 children)

	Outcome: Change in social skills-t at W2				
Predictors	*β* *(SE)*	CI [2.5%, 97.5%]	*p*	TCD	BIC
Model 0 (null model)	<0.01 (0.14)	−0.27, 0.27	1.000	0.00	425.34
Model 1				0.01	428.01
Intercept	<0.01 (0.13)	−0.27, 0.27	1.000		
Child age	−0.16 (0.14)	−0.43, 0.12	0.260		
Model 2				0.30	414.92
Intercept	<0.01 (0.11)	−0.23, 0.23	1.000		
Child age	−0.17 (0.12)	−0.42, 0.07	0.163		
Microaggressions at W1	−0.39 (0.12)	−0.63, −0.15	0.002		
Child–teacher relationship quality at W1	0.47 (0.12)	0.23, 0.71	<0.001		
Model 3				0.59	386.41
Intercept	−0.09 (0.09)	−0.26, 0.08	0.299		
Child age	−0.16 (0.09)	−0.35, 0.02	0.082		
Microaggressions at W1	−0.25 (0.09)	−0.43, −0.06	0.010		
Child–teacher relationship quality at W1	0.35 (0.09)	0.16, 0.53	<0.001		
Microaggressions at W1 * Child–teacher relationship quality at W1	0.59 (0.09)	0.40, 0.77	<0.001		

Model 3 best fit the data, with both highest TCD and lowest BIC. *CI* Confidence interval; *BIC* Bayesian information criterion (Schwarz, 1978); *TCD* Total coefficient determination.

## Discussion

During the school years, children of sexual minority parents may not experience overt bullying from peers related to their family structure; however, they are likely to encounter daily microaggressions in the form of subtle and brief verbal, behavioral, and environmental indignities. If repeated and unaddressed, such microaggressions may undermine child psychosocial adjustment. Middle childhood is a developmental stage in which peers form a central role in children’s immediate social context and children begin to show greater diversification of attachment networks (Seibert & Kerns 2009). This implies that, at school, they may be more likely to use teachers as temporary or ad hoc attachment figures, when upset or worried. Therefore, the current study combined microaggression (Sue & Spanierman 2020), attachment (Verschueren 2015), and developmental intergroup (Bigler & Liben 2006) frameworks to investigate the moderating role of child–teacher relationships in the association between children’s experiences of peer microaggressions and parent- and teacher-rated social skills amongst school-age children of sexual minority parents through assisted reproduction.

Preliminarily, no differences were found in microaggression intensity, child–teacher relationship quality, or social skills on the basis of child gender or family type. On a descriptive level, approximately two-thirds of the children of lesbian mothers and gay fathers reported at least one peer microaggression; and, on average, microaggressions were of a low intensity. Also, child–teacher relationships were, on average, of high quality and characterized by high safe haven–seeking and secure base use, low conflict, low antagonism, and low criticism. Additionally, both parents and teachers reported children to display high levels of social skills. However, children reported that peers asked them intrusive and repetitive questions about having sexual minority parents or being born through assisted reproduction and, in some cases, made assumptions about their family experiences.

Though such microaggressions may seem relatively innocuous compared with overt discrimination, they are not: stressors do not need to reach a traumatic level to produce distress; rather, even daily life hassles, experienced over time, can be stressful (Sue & Spanierman 2020). In this vein, the present results are aligned with previous research on the detrimental impact of microaggressions (Bos & van Balen 2008, Bos et al. 2008, Farr et al. 2016b, Kosciw & Diaz 2008) and the importance of child–teacher relationships (Baker 2006, Demol et al. 2021, Howes et al. 1994, Huang et al. 2018, Murray-Harvey & Slee 2010, O’Connor et al. 2011, Pianta & Stuhlman 2004, Rasheed et al. 2020) for child psychosocial adjustment, showing that more intense microaggressions and lower child–teacher relationship quality predict worse child social skills after 18 months.

Looking more closely at the results, two significant interactive pathways emerged, partially reflecting that children with a lower child–teacher relationship quality had fewer social skills with peers (and vice versa). The first pattern indicated that, as expected, more intense microaggressions predicted lower social skills among children who reported a lower child–teacher relationship quality. From an ecological perspective (Bronfenbrenner 2001), this is straightforward to explain, insofar as children’s relational models (e.g., their relationships with teachers) and experiential context (which may include microaggressions) interact and jointly influence child development. However, also in the context of more intense microaggressions, parents and teachers reported greater social skills in children who reported a higher child–teacher relationship quality. From an attachment perspective, this suggests that, in the school setting, the child–teacher relationship presents similar attachment components to the more enduring and dominant child–parent relationship (Verschueren 2015).

Particularly this second result supports the child–teacher relationship as a potentially secure context in which children can “mentalize” negative experiences such as microaggressions and improve their social skills. However, this relational container may not be utilized to the same extent by children who experience less intense microaggressions. More specifically, and consistent with the key tenets of attachment theory (Bowlby 1988), more vulnerable children (i.e., those experiencing more intense microaggressions) who perceive low conflict in the relationship with their teacher may rely on their teacher as both a safe haven for discussing and elaborating upon microaggressions, and a secure base from which to explore their social environment (i.e., peers). Such children are likely to be more socially competent in terms of, for example, communicating with and inviting peers to join in activities, or responding appropriately to teasing.

The current study has a number of strengths, including its mixed-method, multi-informant, 18-month, and two-wave longitudinal design, as well as the consistency of ratings across parents’ and teachers’ reports of child social skills. The collection of firsthand data on peer microaggressions and the child–teacher relationship from children’s perspectives further provided a more accurate picture of children’s internal and implicit experiences, which parents and teachers are less able to observe. Similarly, the coding of peer microaggressions by two external observers likely minimized social desirability bias and the influence of respondents’ personality traits, which have been limitations of prior microaggression research (Lilienfeld 2017). In addition, while some evidence is available on microaggressions and their impact on behavioral adjustment among adopted school-age children of sexual minority parents in the United States (Farr et al. 2016a, Farr et al. 2016b), no comparable research has examined similar experiences within other diverse family forms of sexual minority parents. Thus, the current study represents a unique contribution to the literature, as it investigated the role of child–teacher relationships in the association between peer microaggressions and social skills among school-age children born to lesbian mothers through donor insemination and gay fathers through surrogacy.

Notwithstanding these strengths, several limitations are also notable. First, the relatively small sample size did not allow separate analyses to be run for children of lesbian versus gay parents, or for female and male children; however, no differences were found across family type and child gender on any of the study variables. A second limitation for generalizability is that the participating families were recruited using convenience sampling, and were overall well-adjusted (Carone et al. 2018, Carone et al. 2020b). Third, research with young children is generally difficult, due to their limited vocabulary, comprehension, and attention span. However, the research team was trained to respond to children’s cues of discomfort in the interviews and to not request expansive responses when these cues appeared. Because the term “microaggression” was likely unfamiliar to the children, they were not directly asked to describe microaggression experiences or to explicitly discuss the intensity of each comment and behavior they referenced. It is possible that, if specifically asked, they might have provided new or different insights about these microaggressive peer interactions or their intensity levels.

Further limitations include the wide middle childhood age range, which prevented a deep exploration of the role of child age in facilitating children’s cognitive recognition and affective coping with microaggressions (given their sometimes ambiguous nature), recourse to teachers during times of distress, and interactions with peers. However, the positive significant correlations that were found between child age and these variables provide a preliminary indication in this direction. Finally, the study did not consider children’s positive conceptualizations and affirmative experiences of their sexual minority parent family; rather, it restricted its investigation to factors representing burdens for the development of children’s social skills. More research incorporating a strengths-based lens (Vaughan & Rodriguez 2014) on the experiences of sexual minority parent families is needed.

Most importantly, the results highlight several implications for theory and practice. As the number of families with sexual minority parents is increasing worldwide, children are more likely to encounter diverse family structures in their daily lives. This is true in Italy, despite the exhausting challenges and barriers that sexual minority people encounter in their journeys to form a family (Lingiardi & Carone 2016a, Lingiardi & Carone 2016b, Scandurra et al. 2019). In light of the prevailing negative attitudes towards sexual minority parent families (rooted in traditional gender ideologies; Ioverno et al. 2018, Pistella et al. 2018), alongside evidence and indications from development intergroup theory that ubiquitous societal stigma may become socialized and internalized among children at an early age (Bigler & Liben 2006, Farr et al. 2019), the implementation of inclusive curricula, anti-bullying policies, and safe-school practices (e.g., gay–straight alliances), with particular reference to sexual minority parent families, as well as the use of children’s books that are more inclusive of family diversity, may contribute to reducing family-related peer microaggressions. In parallel, future studies aimed at understanding how children perceive children of sexual minority parents from a young age could represent a further step towards addressing and minimizing family-related microaggressions (Farr et al. 2019).

Additionally, considering the 18-month longitudinal design adopted in the current study, a follow-up analysis of these children as they enter adolescence could verify the negative effects of microaggressions on social skills and identify further protective factors over time, as children’s social worlds expand, their self-regulating capacities become more sophisticated, their teachers become less relied on as a source of comfort, and their peers increasingly step in to fulfill the role of safe haven (Verschueren 2015). This point is especially crucial, as school-age children may be too young to feel comfortable disclosing microaggressions to others, or they may downplay these experiences as a way of coping (Farr et al. 2016a). Therefore, future research with adolescents of sexual minority parents is recommended to inform a more comprehensive interpretation of microaggressive interactions.

A second implication pertains to the vital role of teachers in supporting—and possibly protecting—microaggressed children of sexual minority parents. As the current results highlight, in their role as ad hoc or temporary attachment figures, teachers must be attuned to the school experiences of children with sexual minority parents and cultivate caring classroom environments that heighten children’s sensitivity to family diversity. To this end, teachers should become aware of their bionormative assumptions about the family, identify microaggressions in the school setting, and construct ways for children to curb and resolve such indignities. Additionally, their direct involvement in attachment-based programs may better equip them to provide appropriate and effective emotional support to children, when needed.

Finally, sexual minority parents must play a key role in this process by preparing children to navigate stigmatizing experiences with resilience and forming positive family conceptualizations (Shenkman et al. 2022). They may do so, for example, by ensuring age-appropriate open communication about their child’s family background; discussing how best to handle questions, comments, and teasing; referring to print and digital media incorporating diverse family forms; and maintaining frequent contact with other children of sexual minority parents (Farr et al. 2016a, Goldberg et al. 2016, Oakley et al. 2017, Wyman Battalen et al. 2019).

## Conclusion

The development of competence in peer interactions has been identified as a key developmental task for school-age children, with profound implications for children’s psychosocial adjustment, school engagement, and broader sense of relatedness, connectedness, and belonging. By mid-elementary school, individual differences in children’s social skills appear to stabilize and predict future adaptive or non-adaptive behavior in adolescence. Also, stigmatization by peers may seriously threaten early adolescents’ well-being. At school, however, peers may inadvertently microaggress children of sexual minority parents through the use of heterosexist terminology, the endorsement of heteronormative ideas of family, and/or direct questioning about the child’s family structure or donor/surrogacy conception. The present findings indicate that school-age children of sexual minority parents living in Italy suffered from low intensity peer microaggressions, and approximately two-thirds experienced at least one microaggression. Furthermore, the findings emphasize that, when these children experienced their teachers as warm, emotionally supportive, non-conflictual, and non-criticizing, they experienced their social environment as trustworthy and less threatening and, in turn, were able to interact with peers safely and competently, even in the face of microaggressions.
