# Correction to: Peer Microaggressions and Social Skills among School-Age Children of Sexual Minority Parents through Assisted Reproduction: Moderation via the Child–Teacher Relationship

**DOI:** 10.1007/s10964-023-01932-1

**Published:** 2023-12-29

**Authors:** Nicola Carone, Eleonora Innocenzi, Vittorio Lingiardi

**Affiliations:** 1 Department of Brain and Behavioral Sciences, University of Pavia, Piazza Botta 11, 27100 Pavia, Italy; 2 Department of History, Culture and Society, “Tor Vergata” University of Rome, Via Columbia 1, 00133 Rome, Italy; 3 Department of Dynamic and Clinical Psychology, and Health Studies, “Sapienza” University of Rome, Via degli Apuli 1, 00185 Rome, Italy

Correction to: Journal of Youth and Adolescence (2022) 51:1210–1229

10.1007/s10964-022-01588-3

In the original publication of the article, the author noticed errors in Table 3. The errors were inadvertently made when cutting and pasting the analytic output into the table. The corrected Table 3 has been presented with this correction.

**Table 3:** Means and standard deviations of peer microaggressions and child–teacher relationship quality at W1, and social skills at W1 and W2, by family type and child gender (*N* = 70 families)

	Lesbian mother families			Gay father families		
	Total (*N* = 37)	Male children (*n* = 19)	Female children (*n* = 18)	Total (*N* = 33)	Male children (*n* = 15)	Female children (*n* = 18)
	*N* (%)	*n* (%)	*n* (%)	*N* (%)	*n* (%)	*n* (%)
Peer microaggressions frequency at W1						
No microaggressions	11 (29.7)	4 (21.1)	7 (38.9)	11 (33.3)	6 (40.0)	5 (27.8)
Yes microaggressions	26 (70.3)	15 (78.9)	11 (61.1)	22 (66.7)	9 (60.0)	13 (72.2)
	*M* (SD)	*M* (SD)	*M* (SD)	*M* (SD)	*M* (SD)	*M* (SD)
Peer microaggressions score at W1^a^	1.30 (1.04)	1.48 (1.05)	1.10 (1.03)	1.24 (1.08)	1.20 (1.15)	1.27 (1.05)
Child–teacher relationship quality at W1	50.24 (11.71)	50.58 (13.49)	49.89 (9.87)	47.09 (12.08)	45.93 (11.84)	48.06 (12.54)
Social skills at W1 (parent ratings)	57.36 (11.79)	59.45 (11.86)	55.17 (11.47)	59.58 (10.94)	59.93 (11.52)	59.28 (10.59)
Social skills at W1 (teacher ratings)	42.86 (6.72)	45.07 (7.42)	40.64 (5.30)	44.07 (6.40)	45.50 (6.24)	43.24 (6.53)
Social skills at W2 (parent ratings)	59.15 (12.11)	60.66 (12.54)	57.56 (11.58)	62.33 (11.45)	63.10 (12.67)	61.69 (10.45)
Social skills at W2 (teacher ratings)	44.86 (11.15)	46.43 (10.32)	43.29 (12.10)	45.59 (12.71)	44.60 (12.38)	46.18 (13.24)

^a^For each child, a single peer microaggression score was calculated by dividing the sum of the intensity of each microaggression by the number of microaggressions reported. Thus, the peer microaggression score reflected both the intensity and the frequency of children’s microaggressions. Percentages may not equal 100 due to rounding. For teacher-rated social competencies at W2, *N* = 55

